# Remarks upon a Method of Dividing Impermeable Strictures within the Canal of the Urethra; Accompanied with Cases

**Published:** 1827-10

**Authors:** R. A. Stafford


### STRICTURES.

*Remarks upon a Method of dividing Impermeable Strictures within the Canal of the Urethra; accompanied with Cases.* By R. A. Stafford, Esq.

Various methods have been employed for the relief of those strictures of the urethra which have been rendered impermeable by thickening and induration, with consequent contraction and irregularity of the canal. The modes of practice which have at different times been resorted to, are forcing a conical sound through the contraction, effecting a passage by the application of caustic, and making an incision through the perineum down to the stricture, and then dividing it. But experience shows that these plans of treatment are more or less liable to objection; that they sometimes aggravate the disease which they are intended to relieve. The caustic is uncertain in its efficacy, often causing considerable constitutional derangement, and even total retention of urine. The attempt to force through the stricture with a conical sound not only brings on, in many cases, inflammation, suppuration, and retention of urine, but is attended with great risk of making a false passage; and the operation of dividing the stricture in the perineum is so little susceptible of being reduced to fixed rules, that it can hardly become a measure of general adoption.

In considering the imperfections of these modes of treatment, it appeared to me that a method could be devised by which the strictured part might be divided within the canal of the urethra, and thus supersede an operation painful in its nature, and of doubtful result. Such a method would effect with certainty, and in a short time, what the caustic is intended to accomplish by repeated and tedious applications. It would prevent the necessity of performing the operation for the incision of the stricture through the perineum, thereby saving the patient the inconvenience and misery of a new channel, and leaving but little for nature to repair; and it would at the same time allow the urine to flow through its natural channel.

With a view of trying to effect such division, I constructed an instrument of a very simple kind.* It consists of a silver catheter, and a stilette with a lancet point, which projects from its handle about half an inch, and is maintained in this position by a spiral spring. On pressing the end of the stilette, the lancet (which is about the eighth of an inch in length,) is thrust forward through the point of the catheter, and recedes into it on withholding the pressure. The outside of the catheter is graduated.

Before using the instrument, the exact distance of the stricture from the extremity of the urethra should be ascertained by the bougie. The armed catheter is then passed down till the point rests against the stricture, (which is known by means of the graduation,) and, being held securely in such position, the spring is pressed by the thumb gently and gradually. As soon as any impression is made, the lancet should be allowed to retire into its sheath, and the blunt point of the catheter urged cautiously forward. If it do not pass on, the lancet may be again used as before. After the stricture is permeated, the armed catheter should be withdrawn, and its place supplied by one of elastic gum, of the same size. This should remain for a day or two, to prevent the union of the divided parts, and to preclude the possibility of extravasation of urine; and, on its removal, a bougie should be passed twice in the week, or as often as may be judged necessary, for some time; and the same treatment adopted as for strictures in general.

In the cases in which the instrument has been employed, the division of the stricture has been followed by more or less inflammation. Such an occurrence should be guarded against by the application of leeches to the perineum immediately after the operation, and by strict adherence to the antiphlogistic regimen. If the presence of the catheter cause considerable pain, it must be withdrawn; but in this case it is of

* The maker of this instrument was Mr. Riley, of Little Britain.

material consequence to pass a bougie daily for a short period, lest the divided parts reunite. Where the stricture also has been so extensive as to be incapable of division by one operation, the same plan should be adopted, in order to maintain the ground already gained.

It may be objected to the use of this instrument, that there is a possibility of making by it a false passage. This is no doubt true; but with care, and by paying attention to the anatomy of the urethra, there is not so great danger of this occurrence as might be imagined, for the lancet is too short to divide much at one puncture, and the end of the catheter too blunt to be forced farther than the incision made. At all events, it is less liable to be forced out of the course of the urethra than a conical sound, and is a more harmless instrument, inasmuch as it divides the parts instead of tearing them asunder. To divide strictures in this manner must certainly be far preferable to the application of caustic, which, as before stated, is uncertain, tedious, and often productive of ulceration of the urethra. It might, perhaps, also be thought that considerable hemorrhage would follow the division of the stricture by this instrument, and that it would produce much pain. But, in the cases in which it has been employed, the bleeding has been inconsiderable, and the pain trifling.

The cases in which the armed catheter appears likely to be most beneficial are those where total or partial retention of urine has arisen from strictures of an indurated and almost a cartilaginous hardness, and which, from this cause, cannot be permeated by any other instrument. It may be useful also in those impermeable strictures which only occupy a short extent of the canal; and in those likewise which are situated above the membranous portion of the urethra. In strictures, however, which are complicated with enlargement of the prostate gland, or are of great extent, and have become tortuous in their course, there is not much likelihood that benefit will arise from its employment.

From the experience which I have now had of its use, I am inclined to hope that the armed catheter may in some cases supersede the necessity of more serious operations, and in others afford relief with more certainty and less delay than the means at present employed. Nothing, however minute, can be considered trifling which has for its object the alleviation of human suffering: I have therefore thought it worth while to publish the result of my own experience of this instrument, in order that those who have the opportunity of giving it a trial may decide upon its utility.

Case I.—George Edwards, ætat. twenty-five, admitted into St. Bartholomew's Hospital, May 12th, 1826, with Stricture of the Urethra, under the care of Mr. Lawrence.

About five years ago he first found the stream of his urine diminished in size, from which time until the present it has gradually become smaller and smaller. He has for the last eight months been incapable of voiding it in any other manner than drop by drop, during which time also he has had frequent attacks of retention of urine, accompanied with hernia humoralis. He suffers much pain in the region of the pubes, has frequent desire to make water, and there is a deposit of mucus in his urine. His tongue is foul, pulse quick, and his general health much impaired. When his urethra was examined, an impermeable stricture was discovered at the distance of five inches and a half from the orifice at the glans penis.

He was ordered milk diet, and placed on a strict antiphlogistic regimen. Leeches were applied frequently to the perineum, occasional warm baths used, and the smallest-sized bougies of every description passed as far as the stricture twice in the week, with the hope of permeating it.

June 5th.—All these means having failed, the armed catheter was introduced as far as the stricture, and used as before described. Three-fourths of an inch of the stricture were divided, and, as this was the first time of using the instrument, no farther attempt was made. As it was thought bougies might tend to keep up inflammation, their use was for the present discontinued. In other respects the case was treated as before.

12th.—On the first day after the operation, he made water in a larger stream than he had done for some time past, but on the following day much worse: indeed, for a few hours he had total retention. For this reason the use of the armed catheter was nearly being abandoned, but, as it was suggested that the man might have become worse, owing probably to the reunion of the divided surfaces of the stricture, Mr. Lawrence gave the instrument another trial. Accordingly, on the following day, its use was had recourse to again, and it was found, as suggested, that the divided portion of the stricture had closed. This, with a very little force, and without pushing out the lancet, was separated. An inch more of the urethra, with the aid of this instrument, was passed. A bougie was introduced every day, to prevent the divided part from closing; when, on Friday the 16th, the armed catheter was again employed, and, with one slight puncture with the lancet, passed with the greatest facility into the bladder. It was then withdrawn, and a silver catheter (No. 9,) was introduced instead of it, and a pint and a half of urine drawn off. He was ordered to keep constantly in bed, and to foment the perineum.

17th.—The catheter caused so much irritation that he could not keep it in the bladder longer than six hours. He had suffered some pain in the perineum, but had slept during the night.

He was ordered to apply twelve leeches to the perineum, to take an aperient draught, and continue fomenting.—The catheter was passed, and withdrawn.

18th.—Had not suffered so much pain; slept tolerably well; and had had his urine drawn off twice without difficulty. Pulse eighty; urine thick, and a sediment of mucus at the bottom of it. The draught had operated three or four times.

To continue fomenting the perineum.

19th.—Had a restless night; felt pain in the perineum, but more in the region of the pubes, and the glands of the groin were swollen. His urine has been drawn off twice, however, without difficulty.

To apply twenty-four leeches,—twelve to the perineum, and twelve to the groin. To take five grains of Calomel and twelve of Jalap immediately. Constantly to foment the perineum and region of the pubes.

Any further account of the case I was prevented from obtaining personally, in consequence of indisposition. I afterwards learnt, however, that in a few days the irritation of the urinary organs subsided, and that the man went away nearly cured; but, having discharged himself from the hospital, any further progress in his case was prevented. At this period, however, a catheter (No. 10) could be easily passed, and he suffered no pain whatever.

Case II.—John Predam, ætat. forty-seven; February, 1827. Has been afflicted with stricture of the urethra for about five or six years, during which time he was admitted as a patient into various hospitals and dispensaries, sometimes with and sometimes without relief, but he has never been completely cured. He now suffers much pain in voiding his urine, which passes from him drop by drop, and he has not been able to have the smallest-sized bougie passed into the bladder for the last six months. In other respects he is in perfect health. Upon examination of his urethra, it was discovered that he had a stricture seven inches from the orifice, and, frequent attempts having been made to pass a bougie through it without success, it was resolved to divide it with the armed catheter.

Feb. 14th.—Having ascertained the exact distance of the stricture from the extremity of the urethra, the armed catheter was passed down to it, and it was divided without much difficulty, with but little pain and slight hemorrhage. There was then substituted a silver catheter of the same size, which was left in the bladder.

He was ordered to take an aperient draught, to foment the perineum constantly with hot water, and to remain in bed.

15th.—He experienced so much pain that he could not keep the catheter in the bladder longer than eleven hours, when it was taken out. He has had almost a sleepless night, but, since the removal of the catheter, has not felt much pain in the perineum. Pulse ninety, and skin rather hot.

Apply twelve leeches to the perineum; to continue fomenting.—The catheter was again passed, and left in the bladder.

16th.—The catheter had remained in the bladder since its last introduction, but its presence had caused considerable pain, which he attributes to its want of pliability, and its striking, when he moves, against the coats of the bladder. It therefore was withdrawn altogether. Pulse ninety-four; skin still hot; no sleep, and there was a sediment of mucus in his urine.

To take an opening draught; to apply twelve leeches to the perineum, and to continue fomenting.

17th.—The pain in the perineum had nearly subsided, and he had made water twice without difficulty. The catheter was passed into the bladder with the greatest ease, and was immediately withdrawn. As the draught had not operated sufficiently, he was ordered to repeat it. Pulse eighty-four; slept better; skin cooler.

18th.—Still less pain in the perineum. His bowels had been relieved freely, and he had slept well during the night; pulse seventy-six; skin cool.

The catheter was again introduced.

19th.—Much better: feels pain only when micturating. Catheter passed without trouble. Pulse seventy-four; skin cool.

20th.—Still feels slight pain while voiding his urine, but all other unpleasant symptoms have vanished.

Catheter was passed, and he was ordered another aperient draught.

21st.—Free from pain altogether; feels himself quite well. He makes water without pain, and in a good-sized stream.

For about a month after this period, a large-sized catheter (No. 12) was passed into the bladder, without the slightest impediment, twice in the week. He now voids his urine with the greatest freedom, in as large a stream as natural, and without the least pain.

Case III.—April 4th, 1827.—Michael Barry, ætat. fifty-one. This man had been suffering so long with stricture of the urethra, that he could not recollect when he first observed the diminution of the size in the stream of his urine. At present, and for the last two months, however, he has been only able to pass his urine drop by drop, and then with so much pain that his life has been rendered quite miserable to him. Many trials have been made to pass bougies of all sizes, from the largest to the smallest, without avail. Upon measurement, the stricture was found to be situated three inches and a half from the glans penis. The armed catheter was introduced to that part, and the stricture divided, with but little pain and slight hemorrhage. The instrument was then withdrawn; and, as it was discovered that another stricture existed in the membranous portion of the urethra, which would not admit of the passage of a catheter larger than No. 5, it was judged necessary not to introduce the gum catheter, but only to pass a bougie through the divided stricture every day. This was done for a fortnight, without the least inconvenience, and the other stricture was treated in the usual manner. In about six weeks both were cured, and the man can now make water as well as ever he did in his life.

During the treatment, leeches were applied to the perineum; purgatives given as often as the symptoms demanded them; and the antiphlogistic regimen strictly adhered to.

*London; August 13th, 1827.*

